# The interplay between intestinal bacteria and host metabolism in health and disease: lessons from *Drosophila melanogaster*

**DOI:** 10.1242/dmm.023408

**Published:** 2016-03-01

**Authors:** Adam C. N. Wong, Audrey S. Vanhove, Paula I. Watnick

**Affiliations:** Division of Infectious Diseases, Boston Children's Hospital, Harvard Medical School, 300 Longwood Avenue, Boston, MA 02115, USA

**Keywords:** *Drosophila melanogaster*, Commensal, Metabolism, Microbiota, Pathogen

## Abstract

All higher organisms negotiate a truce with their commensal microbes and battle pathogenic microbes on a daily basis. Much attention has been given to the role of the innate immune system in controlling intestinal microbes and to the strategies used by intestinal microbes to overcome the host immune response. However, it is becoming increasingly clear that the metabolisms of intestinal microbes and their hosts are linked and that this interaction is equally important for host health and well-being. For instance, an individual's array of commensal microbes can influence their predisposition to chronic metabolic diseases such as diabetes and obesity. A better understanding of host–microbe metabolic interactions is important in defining the molecular bases of these disorders and could potentially lead to new therapeutic avenues. Key advances in this area have been made using *Drosophila melanogaster*. Here, we review studies that have explored the impact of both commensal and pathogenic intestinal microbes on *Drosophila* carbohydrate and lipid metabolism. These studies have helped to elucidate the metabolites produced by intestinal microbes, the intestinal receptors that sense these metabolites, and the signaling pathways through which these metabolites manipulate host metabolism. Furthermore, they suggest that targeting microbial metabolism could represent an effective therapeutic strategy for human metabolic diseases and intestinal infection.

## Abstract (precis)

***Drosophila* Collection:** *Drosophila melanogaster* is an ideal host in which to dissect the impact of intestinal microbes on host metabolism.

## Introduction

Shortly after a sterile gestation and birth, our intestines become colonized by non-pathogenic intestinal microbes that we term our commensal microbiota. These are not a random assortment of organisms but rather a diverse community of microbes that coexist and, under ideal circumstances, maintain a mutualistic, symbiotic relationship with us. Periodically, this community is disrupted by infection and/or antibiotic treatment. Invasion of the intestine by pathogens might edge out members of the commensal microbiota by competing for certain intestinal niches, by creating conditions within the intestine that do not favor commensal growth, or by activating a non-specific innate immune response. Antibiotic treatments, which few of us escape during our lifetimes, are intended to target a particular pathogen, but invariably result in collateral damage within the intestinal microbiota owing to lack of specificity (Fouhy et al., 2012; Relman, 2012; Monira et al., 2013). In the absence of transplantation of intestinal microbiota harvested from a healthy host, restoration of equilibrium within the disrupted microbial community can be delayed by weeks or even months (Reid et al., 2011; Relman, 2012; Nobel et al., 2015; Stewardson et al., 2015).

Derangements of our intestinal microbiota can impact growth and development in childhood and contribute to the pathophysiology of chronic metabolic diseases such as diabetes and obesity (Subramanian et al., 2014; Miele et al., 2015). Although the complex and changing conditions that determine the fluctuations of the intestinal microbial community over a lifetime are poorly understood, mounting evidence suggests that the ability to sculpt the intestinal microbial community could lead to new therapeutic modalities for the prevention of chronic metabolic diseases and intestinal infection. To understand host–microbe interactions in mammals, investigators must consider the complex, interdependent metabolic pathways of the host and the trillions of microbes residing in the intestine. In contrast, the simpler microbiota and signaling systems of the fruit fly *Drosophila melanogaster* as well as the genetic tools available for use in this model organism present the investigator with a unique opportunity to test hypotheses regarding the impact of commensal and pathogenic intestinal microbes on host metabolism in a more controlled and targeted fashion.

Here, we will review what has been learned from the *Drosophila* model about manipulation of host metabolism by both the commensal microbiota of the intestine and intestinal pathogens. Specifically, we will discuss the contributions made by *Drosophila* researchers in elucidating the key metabolites secreted by intestinal commensals and pathogens, host sensing of these metabolites, and the endocrine signals released from the host intestine in response to these metabolites.

After comparing the anatomy and metabolic regulatory pathways of the *Drosophila* and mammalian intestines, we will review in turn studies elucidating the impact of commensal bacteria, bacterial pathogens and viruses of the intestine on host metabolism. Taken together, these studies show that intestinal microbes manipulate host metabolism and, furthermore, that the metabolisms of the host and its intestinal inhabitants are intricately intertwined. Small changes in microbial metabolism can result in large fluctuations in host metabolic homeostasis. We propose that this knowledge can be exploited in the design of prebiotic and probiotic therapies to treat metabolic disease and mitigate the metabolic derangements caused by intestinal infections.

## Comparison of the *Drosophila* and mammalian intestines and their associated metabolic signaling pathways

### Intestinal structures and cell types

**Fig. 1.**
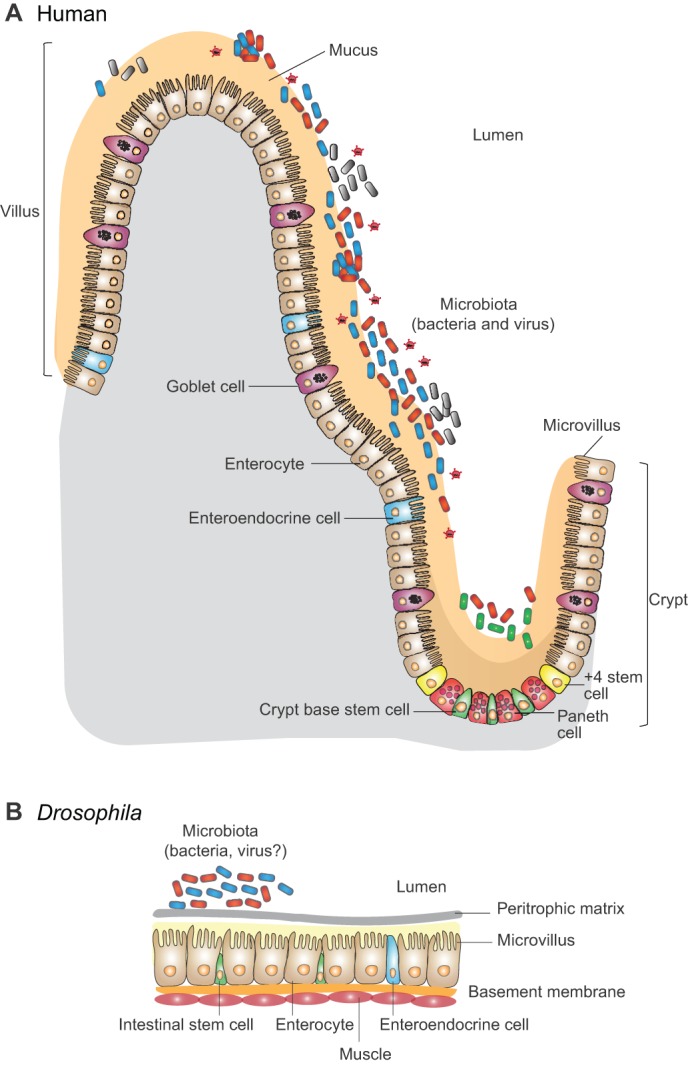
**A** **comparison of the mammalian and *Drosophila melanogaster* intestines.** (A) The contour of the mammalian intestinal epithelium consists of peaks and valleys termed villi (singular: villus) and crypts, respectively. Several cell types with distinct functions are found within the epithelium. Enterocytes, whose surface area is maximized by numerous protrusions known as microvilli, are principally responsible for nutrient absorption. Goblet cells, which are distributed throughout the epithelium, secrete the protective mucus layer composed of polysaccharides and proteins that covers the epithelial surface. Located in crypts, enteroendocrine cells secrete small bioactive peptides in response to signals from nutrients and commensal bacteria in the intestinal lumen. Paneth cells, which are found at the crypt base, secrete antimicrobial peptides and create a stem cell niche. Two stem cell populations are found in the mammalian intestine. Stem cells positioned at the crypt base divide at a constant rate to replenish the epithelium. Division of stem cells located at the +4 position is activated by intestinal insult or infection. The resident commensal microbiota is found within and on top of the mucus layer. (B) The *Drosophila* intestinal epithelium lacks villi and crypts and consists of only three cell types: enterocytes, enteroendocrine cells and stem cells. The peritrophic membrane (or matrix), a structure analogous to intestinal mucus, covers the epithelial surface. The intestinal lumen is colonized by a much less diverse microbiota.

In order to extrapolate from studies of flies to mammals in a thoughtful way, the parallels and differences between the intestines of these two organisms must first be considered. The mammalian intestinal epithelium consists of enterocytes, goblet cells, enteroendocrine cells, Paneth cells and stem cells. These cells are arranged to form protrusions and invaginations termed villi and crypts, respectively (Fig. 1A). Ubiquitously distributed goblet cells are dedicated to the synthesis of the intestinal mucus, a proteoglycan that covers and protects the intestinal epithelium (Birchenough et al., 2015). Enterocytes are principally responsible for absorption of nutrients. To aid with this, their luminal face is lined with small cellular protrusions known as microvilli, which increase surface area.

Nutrients in the mammalian intestinal lumen are sensed by enteroendocrine cells through active transport across the cell membrane or activation of G-protein coupled receptors (GPCRs) on the cell surface (Gribble and Reimann, 2016). Interestingly, some of these GPCRs also function as taste (gustatory) receptors in the mouth (Jang et al., 2007; Dotson et al., 2008; Kokrashvili et al., 2009). For this reason, enteroendocrine cells have been described as the taste organs of the gastrointestinal tract (Sternini et al., 2008; Breer et al., 2012). Activation of these sensors on enteroendocrine cells stimulates the release of small peptide hormones from cytoplasmic vesicles into the local extracellular milieu and systemic circulation. These peptides modulate carbohydrate metabolism, lipid metabolism, intestinal peristalsis and satiety (Drucker, 2001; Naslund and Hellstrom, 2007; Mellitzer and Gradwohl, 2011). Therefore, enteroendocrine cells coordinate local and systemic metabolic responses with the intestinal contents.

The intestinal epithelium is maintained by active and quiescent populations of stem cells (Umar, 2010). The former, positioned at the base of crypts, continually renews the epithelium by producing cells that migrate up to the villus tips and are eventually sloughed off. A second, quiescent, population of stem cells, which is positioned four cells away from the crypt base, divides in response to intestinal insult. Paneth cells, which neighbor the active population of stem cells within the crypt, play a role in maintaining the stem cell niche and also secrete antibacterial peptides and enzymes (Sato et al., 2011; Clevers and Bevins, 2013).

Unlike the mammalian intestinal epithelium, the luminal surface of the *Drosophila* intestine is unconvoluted and comprises only three cell types: enterocytes, enteroendocrine cells and stem cells (Fig. 1B). Only one type of intestinal stem cell has been identified in *Drosophila* (Micchelli and Perrimon, 2006; Ohlstein and Spradling, 2006)*.* These cells replenish all intestinal cell types that are lost as a result of normal senescence or acute intestinal injury (Amcheslavsky et al., 2009; Apidianakis et al., 2009; Buchon et al., 2009; Ren et al., 2010). The *Drosophila* epithelium is covered by the peritrophic matrix, a structure that is analogous to intestinal mucus; this matrix consists of chitin (a polymer of N-acetylglucosamine), and proteins such as peritrophins and drosocrystallin (Lehane, 1997; Kuraishi et al., 2011; Moussian, 2013; Shibata et al., 2015). Enterocytes carry out digestive, absorptive and innate immune functions of the intestine (Marianes and Spradling, 2013; Dutta et al., 2015). In contrast to the wide body of published research elucidating the function of mammalian enteroendocrine cells, *Drosophila* enteroendocrine cells remain relatively unexplored. However, these cells also express gustatory receptors on their cell surface (Park and Kwon, 2011). In addition, they harbor vesicles filled with small peptides that regulate lipid metabolism, carbohydrate metabolism and gut peristalsis (Veenstra, 2009; Song et al., 2014; Vanderveken and O'Donnell, 2014; Kohyama-Koganeya et al., 2015); therefore, they possess all the components required to fulfill the same function as mammalian enteroendocrine cells in coordinating a systemic response to nutrients and metabolites in the gut lumen.

### Control of systemic carbohydrate mobilization and storage by the mammalian and fly intestinal epithelia

Appropriate carbohydrate utilization and storage is important for the maintenance of metabolic homeostasis in all animals and, therefore, is tightly controlled by the endocrine system. In response to ingestion of nutrients, both mammals and *Drosophila* release small peptide hormones termed insulin or insulin-like peptides from specialized cells (Barbieri et al., 2003; Nassel et al., 2013). Insulin receptors on adipose tissue and muscle cells sense these peptides and activate the insulin/insulin-like growth factor signaling (IIS) pathways, which inhibit gluconeogenesis and glycogenolysis and promote glycogen and triglyceride storage (Khan and Pessin, 2002; Teleman, 2010; Padmanabha and Baker, 2014). In mammals, low levels of carbohydrates result in secretion of glucagon, an endocrine peptide that activates glycogen catabolism (Marroqui et al., 2014). In *Drosophila*, adipokinetic hormone seems to fulfill the role of glucagon (Bednarova et al., 2013).

**Fig. 2.**
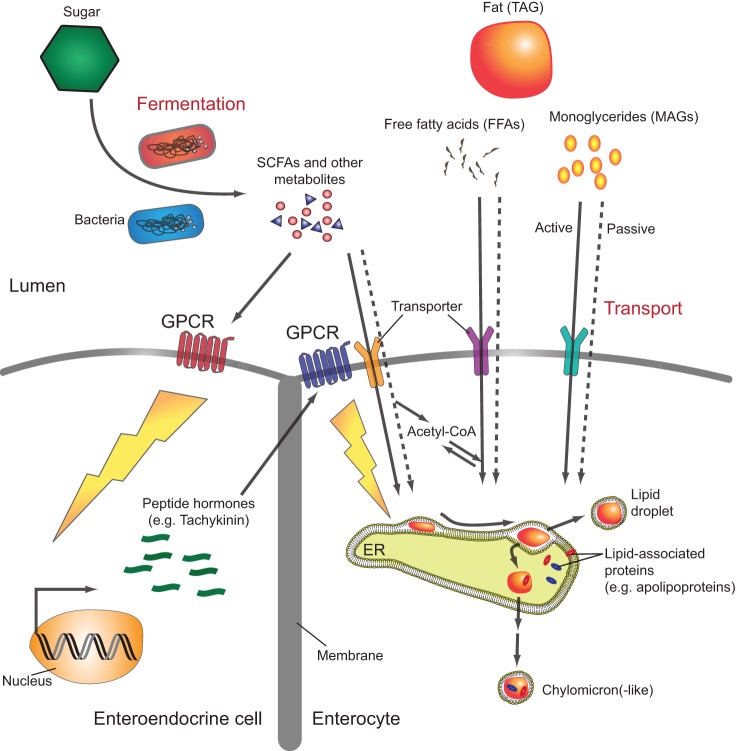
**Signaling pathways through which gut microbiota modulate host carbohydrate and lipid metabolism.** Consumed nutrients are metabolized by the gut microbiota to produce bioactive metabolites that are sensed by the mammalian and possibly the *Drosophila* epithelium. Short-chain fatty acids (SCFAs), the product of bacterial carbohydrate fermentation, and other bacterial metabolites are taken up by enterocytes (note that the villi are not shown in this representation) and converted into metabolically active molecules such as acetyl-CoA, or sensed by specific G-protein-coupled receptors (GPCRs) expressed on the surfaces of enterocytes and enteroendocrine cells. This, in turn, triggers release of enteroendocrine peptides into the systemic circulation and activates signaling cascades that modulate host carbohydrate and lipid utilization both in the intestine and systemically. Dietary triacylglycerides (TAGs) are hydrolyzed into monoacylglycerides (MAGs) and free fatty acids (FFAs) before being absorbed by enterocytes. These lipids accumulate within the leaflets of the endoplasmic reticulum (ER) membrane and are then packaged either into lipid droplets for storage or into lipoprotein particles for transport to other tissues.

In mammals, the commensal microbiota plays a role in the regulation of systemic glucagon and insulin secretion through its production of short-chain fatty acids (SCFAs), whose aliphatic tails contain fewer than six carbon atoms (Shen et al., 2013; Everard and Cani, 2014). Microbial metabolism within the intestine, which results in excretion of acetate [two carbon atoms (C2)], propionate (C3) and butyrate (C4), accounts for 95% of the body's SCFAs. These bacterial metabolites not only serve as nutrition for enterocytes but are also sensed by the GPCRs GPR109A, FFAR2 and FFAR3 on intestinal cells (Fig. 2). GPR109A is expressed in colonic enterocytes, where activation by butyrate suppresses inflammation. Both FFAR2 and FFAR3 are expressed in enteroendocrine L-cells, which secrete regulatory peptides such as protein YY (PYY) and glucagon-like peptide 1 (GLP-1) in response to SCFAs. GLP-1 inhibits glucagon release and promotes insulin secretion, resulting in lowering of blood glucose, whereas PYY reduces appetite (Kasubuchi et al., 2015; Psichas et al., 2015). Thus, bacteria communicate the status of their own metabolism to enteroendocrine cells through the metabolites they produce. The enteroendocrine cells, in turn, appropriately adjust host food intake and metabolism.

Although the regulatory cascade is not as well defined in flies, all indications are that a similar process is at work. The regulatory peptide IMPL2, which is synthesized and secreted by enteroendocrine and possibly other cell types, blocks insulin signaling (Garbe et al., 1993; Marianes and Spradling, 2013; Sarraf-Zadeh et al., 2013; Dutta et al., 2015). It seems to function by directly binding to and inhibiting the function of the *Drosophila* insulin-like peptides (Dilps), which activate the insulin receptor (Alic et al., 2011). Furthermore, evidence suggests that intestinal acetate increases signaling through the insulin pathway by repressing transcription of IMPL2 (Shin et al., 2011; Hang et al., 2014; Kwon et al., 2015). Taken together, these data suggest that a receptor for acetate is present in the *Drosophila* intestine, possibly on the surface of enteroendocrine cells, and that, similarly to the mammalian system, activation of this receptor modulates insulin signaling.

### Lipid uptake and mobilization in the intestine

In mammals, continued ingestion of lipid-laden foods correlates with the development of diabetes and obesity (Moran-Ramos et al., 2012; de Souza et al., 2015), and modulation of the metabolic response to dietary lipids through manipulation of the intestinal microbiota has been proposed as a therapeutic option for these diseases (Cani et al., 2008; Serino et al., 2014). An in-depth understanding of how intestinal microbiota modulate lipid uptake is key to the development of such therapies.

The dietary lipids of mammals are principally composed of triacylglycerols (TAGs). These lipids are emulsified by bile and then enzymatically degraded by pancreatic lipase, yielding free fatty acids (FFAs) and monoacylglycerols (MAGs) in the intestinal lumen (Fig. 2) (Mansbach and Gorelick, 2007). Through as-yet poorly defined pathways, these lipid products are taken up by enterocytes by mechanisms that include both passive diffusion and active transport. The fatty acid transport proteins (FATPs), which are hypothesized to function as acyl-CoA synthases and/or transporters, the fatty acid translocase FAT (CD36) and the plasma-membrane-associated fatty-acid-binding protein FABpm have all been implicated in fatty acid uptake from the intestinal lumen (Mansbach and Gorelick, 2007). Once imported, fatty acids are transported to the endoplasmic reticulum, where they are reassembled into TAGs, which accumulate between the leaflets of the endoplasmic reticulum membrane. These collections of TAGs eventually bud off to form lipid-storage droplets or are packaged into lipoprotein particles, known as chylomicrons, within the ER lumen and released into the lymph (Buttet et al., 2014). In addition to proteins, chylomicrons contain large amounts of TAG as well as cholesterol and fat-soluble vitamins (Iqbal and Hussain, 2009). They are surrounded by a phospholipid monolayer that is principally composed of phosphatidylcholine. Chylomicron-associated proteins as well as the particles themselves are synthesized by enterocytes expressly for transport of dietary fat.

Many aspects of the lipid uptake mechanisms of the *Drosophila* intestine remain largely unexplored. A biliary system is not present in the fly intestine and, although an emulsifying substance might be secreted by enterocytes, none has been identified to date. In fact, one might argue that, because TAG is not abundant in the natural food sources of *Drosophila* (such as rotting fruit), an emulsifying agent analogous to bile is not essential for lipid absorption in this organism. Luminal digestion of TAG is carried out by the Magro protein, a homolog of the mammalian gastric lipase (Sieber and Thummel, 2009, 2012). Absorption of fatty acids from the intestinal lumen has not been studied. However, homologs of FATP, CD36 and FABPpm are all present in the *Drosophila* genome (Adams et al., 2000). Absorbed dietary lipids are presumably then trafficked to the endoplasmic reticulum, where they are either retained in lipid droplets or packaged for transport through the hemolymph to the specialized *Drosophila* adipose tissue known as the fat body for storage, or to other organs for catabolism. Tachykinin, an enteroendocrine-cell-derived regulatory peptide, activates mobilization of lipids from the intestine to the hemolymph (Song et al., 2014). Although the precise mechanism by which Tachykinin acts has not been elucidated, alterations in the transcriptional profile of genes involved in lipid metabolism seem to play a role.

Three lipoproteins – lipophorin (Lpp), the lipid transfer particle (LTP) and Crossveinless D (Cv-D) – have been implicated in systemic lipid transport in *Drosophila* (Palm et al., 2012). Lpp is responsible for the transport of 95% of the lipids carried in the hemolymph, whereas LTP and Cv-D contribute to a much lesser extent. Knockdown of both LTP and Lpp leads to accumulation of lipid droplets in the intestine, demonstrating that these proteins are required for mobilization of intestinal lipid stores and dietary fat (Palm et al., 2012). By facilitating recruitment of Lpp to the Lpp receptor, LTP, in particular, seems to be crucial for the transfer of lipids from the gut to Lpp and for the uptake of lipids by distant tissues (Rodriguez-Vazquez et al., 2015).

Whereas the mammalian proteins dedicated to the transport of dietary lipids are synthesized by enterocytes, Lpp and LTP are synthesized in the fat body and are transported to the intestine. Furthermore, whereas chylomicrons principally contain TAG and phosphatidylcholine, the principal lipids associated with lipophorin in the hemolymph are diacylglycerol and phosphatidylethanolamine (Panakova et al., 2005; Palm et al., 2012). However, the evolutionary and physiological significance of these small differences in lipid transport has not been investigated.

## Commensal microbiota and host metabolism: impact on development and health

**Fig. 3.**
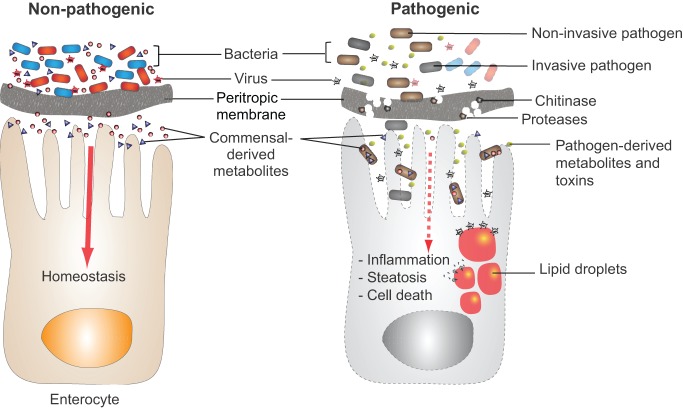
**Pathogens and non-pathogens modulate intestinal metabolism differently.** The metabolites produced by commensal (non-pathogenic) bacteria play a key role in maintaining gut homeostasis, and bacteriophages trim and tailor the bacterial population. The peritropic membrane comprises chitin and proteins. By secreting proteases and chitinases, bacterial pathogens can digest, and thus weaken, the peritrophic barrier, allowing these bacteria to invade the intestinal epithelium. Alternatively, a non-invasive pathogen might interrupt signaling between commensals and the host intestine by consuming commensal metabolites or producing virulence factors that mute host signaling pathways. If intestinal lipid metabolism is dysregulated, the resulting lipid droplets within enterocytes can provide a platform for replication of viruses that exploit these organelles, thus promoting viral superinfection.

A universal feature of organisms with open digestive tracts is colonization of the gastrointestinal tract by a characteristic commensal microbiota. This microbiota, which thrives on the nutrients produced by digestion of the host's diet and intestinal secretions, is shaped by host-specific selective pressures such as the intestinal environment, food preference and eating habits (Ni et al., 2015). In turn, the microbiota manipulates host metabolism by altering nutrient availability, generating essential nutrients, and excreting metabolites that serve as a form of interspecies communication (Fig. 3). As touched upon above, this complex interaction plays a key role in childhood growth and development, and in adult metabolic homeostasis (Ukhanova et al., 2012; Ahmed et al., 2014).

Because of its less-complex and more-tractable microbiota in comparison to mammals, *Drosophila melanogaster* provides a useful model in which to study the governing principles of the host's metabolic interaction with its microbiota. Whereas humans are exposed to the diverse microbiotas that populate a wide array of plant and animal food sources, wild *Drosophila* have a more limited diet of overripe fruits and vegetables, decomposing plants, and fungi. Accordingly, the intestinal microbiota of the wild fly consists of five to 30 taxa (Wong et al., 2011; Broderick and Lemaitre, 2012), as compared with the greater than 500 taxa in the human intestinal microbiota (The Human Microbiome Project Consortium, 2012). The laboratory fly's microbiota is further limited by its artificial environment. The intestinal microbiome of a captive fly consists principally of the microbes expelled by its predecessors into the fly medium (Blum et al., 2013). In spite of this, some bacterial species, such as those belonging to the genera *Acetobacteraceae* and *Lactobacillaceae*, are found in both laboratory-raised and wild *Drosophila* (Cox and Gilmore, 2007). Of course, it is possible that the genomes of the *Acetobacteraceae* and *Lactobacillaceae* found in wild and laboratory-raised flies have diverged in metabolically important ways owing to the distinct selective pressures of the laboratory and natural environments.

The simplicity of the laboratory-raised fly microbiome affords experimental tractability. *Acetobacter* and *Lactobacillus* species, which predominate in the intestines of laboratory-raised flies, are readily cultured and easily eliminated to generate axenic, or ‘germ-free’, animals. Furthermore, comparative studies of development, nutrient allocation and metabolic signaling in conventional and axenic flies suggest that the intestinal microbiota impacts the physiology of flies and mammals in parallel ways.

### The microbiome and development

In the developing world, the microbiota has been shown to influence risk of malnutrition, growth retardation and cognitive delay (Smith et al., 2013; Ahmed et al., 2014; Kane et al., 2015). However, the particular microbes involved and their specific functions in these pathophysiological processes are often difficult to pinpoint. Similar to mammals, the *Drosophila* microbiota is important for normal development. Because the *Drosophila* genome is genetically tractable and less redundant than mammalian genomes, the mechanism by which the microbiome impacts development is better understood. *Drosophila* are generally raised on nutrient media containing a source of sugar and protein as well as yeast or yeast extract. Yeast is a rich source of lipids and B vitamins as well as proteins and carbohydrates. A decrease in the yeast content of *Drosophila* medium has been shown to cause developmental delay due to a reduction in signaling through the target of rapamycin (TOR) pathway, a highly conserved pathway also found in mammals (Layalle et al., 2008; Storelli et al., 2011; Wong et al., 2014). A similar developmental delay was found in axenic flies, and this developmental delay could be rescued not only by supplementation with yeast or B vitamins (Shin et al., 2011; Wong et al., 2014) but also by re-association with *Acetobacter pomorum* (Shin et al., 2011) or *Lactobacillus plantarum* (Storelli et al., 2011). Re-colonization with either of these commensal organisms activated insulin signaling, a process that is central to *Drosophila* development (Nassel et al., 2015).

Highlighting the power of the fly model, Shin and colleagues went on to identify the *A. pomorum* protein pyrroloquinoline quinone-dependent alcohol dehydrogenase (PQQ-ADH) as being required to rescue development, by generating a library of *A. pomorum* transposon mutants and re-associating each mutant singly with an axenic fly population (Shin et al., 2011). Finally, Shin determined that acetate, the product of PQQ-ADH, was the microbial signal that was essential for activation of insulin signaling and normal *Drosophila* development. In another example of the power of the *Drosophila* model, Erkosar et al. recently showed that the beneficial effect of *L. plantarum* on host development is mediated by its activation of host intestinal proteases (Erkosar et al., 2015). This effect could be replicated by intestinal overexpression of one of these proteases in germ-free larvae and negated by infection of *L. plantarum*-monocolonized flies with a pathogen.

Because screens of individual bacterial mutants in mice are labor intensive and costly, technologies such as signature-tagged mutagenesis (STM) and transposon sequencing (Tn-seq) have been developed to assess sub-libraries of 100 or more mutants simultaneously (Shea et al., 1996; van Opijnen et al., 2009). Importantly, using these high-throughput approaches, the presence of a mutant carrying a mutation in a gene such as *PQQ-ADH*, which eliminates excretion of the bioactive metabolite acetate, would be detected neither by the host nor the investigator. Thus, the approach taken by Shin, namely screening of bacterial mutants singly, is the most powerful approach for identification of bacterial genes responsible for the production of active microbial metabolites in the host intestine. Such an approach is accessible to most investigators only when undertaken in a model invertebrate host such as *D.* *melanogaster*, highlighting the potential of this model to accelerate investigations of the role of the microbiota in normal development and developmental disorders*.* In line with the scope of this article, below we focus specifically on insights into the role of the intestinal microbiota in the development of obesity that have been gleaned from studies of the fly intestinal microbiome.

### The microbiota and obesity

In mammals, metabolites secreted by the microbiota modulate adult metabolism as well as juvenile development. For instance, SCFAs regulate appetite, insulin signaling and adipogenesis through specific GPCRs located on enteroendocrine cells, sympathetic neurons and adipose tissue (Ichimura et al., 2009, 2014; Hara et al., 2014). Not surprisingly, therefore, the intestinal microbiota can direct host storage of lipids in adipose tissue, leading to obesity. For instance, specific antibiotic-driven perturbations of the mouse microbiota during development predispose these mice to obesity (Cox et al., 2014). By contrast, when fed a high-fat diet, germ-free mice are less prone to obesity as compared with their conventionally raised counterparts (Rabot et al., 2010). However, in the mouse, the specific microbe or microbes responsible for this effect have not been identified. Experimental manipulation of the *Drosophila* microbiota also suggests a role in nutrient allocation and metabolism. Investigators in the Douglas and Lee labs showed that germ-free flies given access to standard fly medium develop hyperglycemia and hyperlipidemia (Shin et al., 2011; Ridley et al., 2012; Newell and Douglas, 2014; Wong et al., 2014). Recolonization with single bacterial species reversed hyperglycemia, whereas multiple bacterial partners were required to reverse hyperlipidemia (Newell and Douglas, 2014). These studies demonstrate the power of the *Drosophila* model as a platform on which to dissect the effects of distinct intestinal microbes on host metabolism and to experiment with the design of an intestinal microbiota that maximizes metabolic health and minimizes obesity risk.

In humans, genetic background is known to contribute to the development of diabetes and obesity (Bouchard, 1997; Ridaura et al., 2013; Basile et al., 2014). Because the intestinal microbiota also plays a role in the development of these diseases, the role of host genetics in shaping the intestinal microbiota remains a top research priority. In humans, accumulating evidence suggests that the genetic background of the host alters the intestinal microbiota and this, in turn, modulates host metabolism (Goodrich et al., 2014; Zhang et al., 2015). In one study, mice of three different genetic backgrounds with varying susceptibility to obesity and diabetes were bred in a common environment (Ussar et al., 2015). Metabolic phenotypes were found to be the product of not only the environment, but also the host genetic background. However, precise host genes were not implicated. The power and ease of *Drosophila* genetics make this an ideal model for such studies. In a genome-wide association study (GWAS) using the *Drosophila* Genetic Reference Panel (DGRP), Dobson and colleagues demonstrated that host genetic polymorphisms greatly influence microbiota-dependent nutritional phenotypes (Dobson et al., 2015). These studies suggested that a single gene mutation could, in some cases, reverse a microbiota-dependent nutritional effect. However, correlations of nutritional phenotypes with host genotype were evaluated only by testing loss-of-function mutants. Although additional genetic experiments are required to solidify these associations, this work paves the way for future mechanistic studies exploring the impact of host genotype on the host–microbiota metabolic interaction.

The intestinal microbiome affects our well-being by ensuring normal development, adequate nutrition, and appropriate carbohydrate and lipid metabolism throughout life. Therefore, the ability to maintain a microbiome ideally matched to the host would greatly improve human health. Because of the genetic tools available to *Drosophila* researchers and the obvious parallels to the mammalian system, this is an ideal host in which to explore the genetic barriers to the maintenance of the designer microbiome.

## Bacterial pathogens and host metabolism

Anorexia, malaise and diarrhea are all symptoms of intestinal infection. In the developing world, multiple intestinal infections in rapid succession are an important cause of malnutrition, wasting and a general failure to thrive in children under five (Kotloff et al., 2013). Although the rapid transit of nutrients through the intestine that defines diarrhea is bound to be responsible for some of this, research in *Drosophila* has elegantly revealed pathogen impacts on host metabolism that extend beyond decreased intestinal transit time.

*Mycobacterium marinum*, a mycobacterial species that causes skin infections associated with abrasions acquired during water exposure, is sometimes used as a model for *Mycobacterium* *tuberculosis* (Deng et al., 2011). Early work by the Schneider laboratory showed that, when injected into the *Drosophila* hemolymph, this extra-intestinal pathogen caused wasting through dysregulation of insulin signaling (Dionne et al., 2006). The ultimate result was a decrease in glycogen and triglyceride stores along with an elevation in systemic glucose levels, suggesting that systemic infections might cause insulin resistance in *Drosophila* as they do in mammals (Gheorghita et al., 2015). The study by Dionne et al. set the stage for subsequent explorations of the impact of diarrheal pathogens on *Drosophila* metabolism.

It has been shown using flies that systemic infection with the intestinal pathogens *Salmonella typhimurium* and *Listeria monocytogenes*, but not the common intestinal inhabitant *Enterococcus faecalis*, results in anorexia (Ayres and Schneider, 2009). Development of anorexia, in turn, impacts expression of antimicrobial peptides and susceptibility to infection. Thus, the host metabolic state can alter interactions with invading pathogens by modulating the innate immune response. Interestingly, this group also reported that anorexia was induced by decreased expression of the gustatory receptor Gr28b (Ayres and Schneider, 2009), which is highly expressed in enteroendocrine cells (Buchon et al., 2013; Marianes and Spradling, 2013). This gustatory receptor, as well as others expressed on the surface of intestinal cells, provides a mechanism whereby the products of pathogenic intestinal microbes can activate signaling pathways that alter host satiety and susceptibility to infection.

The group led by David Schneider subsequently employed metabolomic studies to demonstrate that *L. monocytogenes* infection decreased glycogen and triglyceride stores as well as the glucose concentration in the hemolymph (Chambers et al., 2012). The group also noted that levels of the anti-oxidant uric acid were decreased. Although these changes are presumably the result of infection-induced anorexia and other bacterial impacts on the host, this study did not conclusively identify these changes in host metabolism as components of a pathogen virulence program, a host innate immune response or a specific host–pathogen interaction pathway.

Because of the speed and affordability of genetics, the comprehensive mutant and transgenic RNA interference (RNAi) lines, and the eminently accessible and extensive databases, the *Drosophila* model is ideally suited to rapid dissection of host–pathogen interactions (Ni et al., 2008, 2011; Cook et al., 2010; dos Santos et al., 2015). Using these tools, subsequent studies have thus far revealed three distinct pathways co-opted by intestinal pathogens to modulate host metabolism. *Pseudomonas entomophila*, a bacterium originally isolated from flies, causes a lethal infection when ingested (Liehl et al., 2006). By contrast, *Pectobacterium carotovorum* strain 15 (*Ecc*15) induces a strong innate immune response when ingested, but does not kill the fly (Basset et al., 2000). Chakrabarti and colleagues compared the transcriptomes of flies infected with these two bacteria and found that a number of stress response genes were selectively activated in *P. entomophila* infection (Chakrabarti et al., 2012). In addition, whereas transcription of antimicrobial peptides was greatly activated by infection, expression of a *diptericin-lacZ* reporter fusion could not be detected, leading the authors to conclude that translation but not transcription was inhibited by *P. entomophila*. The mechanism of translation inhibition was determined to be the result of phosphorylation of elf2α by the GCN2 kinase and inhibition of the TOR pathway, which is known to activate protein translation. The *P. entomophila* pore-forming toxin, monalysin, was found to be at least partially responsible for this block in translation. Although a connection between host metabolism and protein translation in the intestine was not explored in this study, we hypothesize that a burst of transcription and subsequent translation is likely part of the intestinal response to the ingestion of dietary nutrients. When this is blocked by an intestinal pathogen, the host metabolic response might be altered such that intestinal nutrients are not optimally utilized. Therefore, a block in protein translation could be a cause of wasting in particular intestinal infections.

The human intestine has evolved to detect and respond to the metabolic waste products of its commensal bacterial inhabitants, and the importance of the host response to these bacterial metabolites in human health and metabolic disease is just beginning to be appreciated (Canfora et al., 2015). Parallel symbiotic interactions between *Drosophila* and its commensal intestinal microbiota have been identified, although the cell types and receptors that detect these bacterial metabolites have not yet been identified (Shin et al., 2011). We hypothesize that intestinal pathogens contribute to and catabolize metabolites within the host intestine uniquely such that the host response to pathogens is distinct from its response to commensals. However, this aspect of the host–pathogen interaction remains poorly understood and is an area in which *Drosophila* researchers have made and are poised to make seminal contributions.

In *Drosophila*, unique metabolites of intestinal pathogens have been reported to activate the host intestinal innate immune system. In particular, Lee et al. determined that the metabolite uracil secreted by *Ecc*15 is an activator of intestinal transcription of the *Drosophila* gene encoding the reactive-oxygen-generating protein dual oxidase (Duox) (Lee et al., 2013). Based on the signaling pathway mediating *duox* activation, they proposed the existence of a GPCR that responds to uracil (Lee et al., 2015). Furthermore, they reported that this metabolite is also secreted by the intestinal pathogens *Vibrio fluvialis*, *Shigella sonnei*, *Pseudomonas aeruginosa* and *Serratia marcescens* but not by the commensal organism *Commensalibacter intestini* when cultured in minimal medium*.* Notably, *Klebsiella pneumoniae*, which is a normal inhabitant of the human intestine, also produced uracil in culture. Because essential metabolic pathways of bacteria are highly conserved, we propose that commensal bacteria and pathogens could differ primarily in how these metabolic pathways are regulated within the intestinal environment, resulting in differences in the repertoire of metabolites produced. Although the Lee et al. study did not specifically study the effect of Duox activation on host metabolism, we predict that the resulting activation of a non-specific intestinal innate immune response, which decreases the number of pathogenic and commensal bacteria, could result in disruption of host metabolic homeostasis.

Another example of the interaction of host and pathogen metabolisms was discovered by Hang et al. (2014). In this case, acetate, a metabolite normally supplied to the host by the commensal microbiota, was consumed by the intestinal pathogen *Vibrio cholerae*, leading to a decrease in insulin signaling, depletion of lipid stores in the fat body and the appearance of large lipid droplets in enterocytes. This metabolic phenomenon is likely due to the observed transcriptional activation of intestinal IMPL2, an inhibitor of insulin signaling (Honegger et al., 2008). Interestingly, overexpression of IMPL2 has recently been implicated in organ-wasting phenotypes (Figueroa-Clarevega and Bilder, 2015; Kwon et al., 2015). This presents an additional mechanism by which intestinal infection could lead to host wasting.

Manipulation of host metabolism by bacterial pathogens could have the added effect of predisposing the host to viral infection. Recently, the Cherry laboratory has demonstrated that insulin signaling protects *D. melanogaster* against infection by certain viruses, through activation of the MAPK signaling pathway and phosphorylation of ERK (Xu et al., 2012, 2013). This supports the hypothesis that, by suppressing insulin signaling, bacterial infection of the intestine might predispose the host to viral superinfection.

The metabolites of commensal intestinal bacteria are sensed by enterocytes, enteroendocrine cells, and possibly cells of other types in the intestine. The host responds to these bacterial signals by adjusting carbohydrate and lipid metabolism. Wasting in the setting of intestinal infection, which has been documented in both humans and *Drosophila*, is likely to be partially the result of pathogen interference with these ‘conversations’ between the host and its intestinal microbiota. Studies of this phenomenon in *Drosophila* have revealed a variety of mechanisms by which pathogens interrupt this communication (Fig. 3). Intestinal pathogens might secrete toxins that block the host translational response to bacterial signals. They might activate the innate immune response leading to a shift in the commensal population and, therefore, the bacterial metabolites produced by this population. Finally, they might silence the communication by consuming the metabolites secreted by the commensal population. The benefit to the pathogen of silencing the host–commensal communication has not yet been explored. However, these studies suggest the hypothesis that disruption of intestinal nutrient transport and metabolism leaves more dietary nutrients in the intestinal lumen. These nutrients are available to the luminal pathogen to support its growth and replication. In other words, the host wastes while the intestinal pathogen feasts.

## Intestinal viruses and host metabolism

Researchers are just beginning to mine the virome of the healthy and diseased mammalian intestine (Minot et al., 2013; Norman et al., 2015). This virome includes both enteric viruses that target eukaryotic cells and viruses also known as bacteriophage, which target intestinal bacteria. Owing to the requirement for a specific host receptor, bacteriophage cause lysis in a very narrow range of bacterial hosts (Buckling and Brockhurst, 2012; Diaz-Munoz and Koskella, 2014). Therefore, it has been hypothesized that enteric bacteriophage exclude particular microbes from the intestinal milieu, thus shaping the commensal bacterial population (Mills et al., 2013). Because the intestinal microbiota plays a role in regulating host metabolism, it seems inevitable that relationships between the enteric virome and host metabolism will also be established. A similar multi-species interaction is likely to be at play in the *Drosophila* intestine, supporting its use as a model to explore these relationships and their importance in the maintenance of human health.

Many viruses that are pathogenic in humans, including dengue and hepatitis C, use lipid droplets as a platform for viral replication (Saka and Valdivia, 2012). In the case of hepatitis C, infection is associated with an increase in the number of lipid droplets in hepatocytes (Filipe and McLauchlan, 2015). Although the mechanism by which lipid droplets multiply in response to infection has not been elucidated, the virus might use this to amplify its own replication.

A similar virulence mechanism is observed in *D. melanogaster* infection by the flock house virus (FHV), a natural insect pathogen. Infection of *Drosophila* cells with FHV increased transcription of genes encoding lipid metabolism proteins such as the CTP:phosphocholine cytidylyltransferases, Cct1 and Cct2 (Castorena et al., 2010). These proteins, which are found in association with the surface of lipid droplets, are required for the synthesis of phosphatidylcholine from diacylglycerol (Moessinger et al., 2014). RNAi inhibition of Cct1 and Cct2 expression resulted in decreased viral replication, suggesting that FHV might also use lipid droplets as a platform for replication. Although additional studies are required, these examples suggest that use of the lipid droplet surface as a replication platform is a conserved virulence mechanism in viruses that infect both mammals and *Drosophila*.

## Summary and future directions

Although the cast of commensal and pathogenic microbes in the *Drosophila* and mammalian intestines are distinct, the literature reviewed here strongly suggests that the pathways by which these microbes interact with their host intestines are highly conserved. In both, the relevant intestinal structure holds bacteria at a distance from the epithelium, provides a surface for attachment, and serves as a source of nutrition for intestinal bacteria (Ritchie et al., 2010; Kuraishi et al., 2011; Purdy and Watnick, 2011; Hang et al., 2014; Faderl et al., 2015; Tailford et al., 2015). Furthermore, the intestinal epithelium senses and responds to nutrients and microbial metabolites by altering intestinal motility and host metabolism (Drucker, 2001; LaJeunesse et al., 2010; Vanderveken and O'Donnell, 2014).

One important aspect in which these two epithelia differ is in their topology (Fig. 1). Whereas the intestinal epithelium of the fly is flat, the villi and crypts of the mammalian epithelium create invaginations in the epithelial surface that might not be accessible to all intestinal bacteria. Although the factors that enable crypt colonization have not been defined, this ability is characteristic of particular intestinal pathogens (Iimura et al., 2005; Olivier et al., 2007). Because the antimicrobial-peptide-secreting Paneth cells reside at the crypt base, we hypothesize that the high concentration of antimicrobial peptides at the crypt base shapes both the crypt microbiota and susceptibility to pathogen colonization (Shim et al., 2010). Furthermore, because stem cells and enteroendocrine cells reside towards the bottom of these crypts, access of both commensal and pathogenic bacteria to these spaces might impact their influence on renewal of the epithelial surface and host metabolism. Therefore, although the cell types and intestinal signaling pathways that control host metabolism are similar, the proximity of the microbiota to the analogous mammalian host cell must be considered before extrapolating from *Drosophila* to mammals.

The differences in the microbiota of mammals and flies must also be considered when drawing parallels between these two organisms. Whereas the principal members of the fly microbiota are *Lactobacilli*, which are Gram-positive facultative anaerobic rods of the *Firmicutes* phylum, and *Acetobacter* species, which are Gram-negative aerobic rods of the *Proteobacteria* phylum, a much more diverse microbiota is found in the mammalian gastrointestinal tract. However, because each microbiota is exquisitely matched to its host intestine, considerable differences in microbiota are found even when using a mammal, such as the mouse, to model the human gastrointestinal tract (Nguyen et al., 2015). These differences in microbiota do not invalidate the use of non-human models. As we have highlighted in our discussion, studies with model organisms suggest that, although the participants in the conversation are very different, the metabolic dialog itself is quite similar.

In mice and humans, many of the intestinal receptors that sense the byproducts of microbial metabolism have been identified. In *Drosophila*, these receptors are also likely to exist. However, not one has been identified to date. This represents a great gap in our understanding of the interaction of *Drosophila* with its intestinal microbiota, and one that can be rapidly filled given the genetic tools available for this model. We believe that this is one area where future research efforts should be focused.

Microbes that reside within our intestines can both promote and impede optimal nutrient utilization by sharing and catabolizing the nutrients we consume. We share our nutrients with commensal microbes, and they, in return, catabolize indigestible foods, delivering to us digestible byproducts (Hooper et al., 2002; Rakoff-Nahoum et al., 2014). To streamline this process, the eukaryotic intestine has evolved pathways, such as those housed by enteroendocrine cells, to sense and respond to the metabolites produced by symbiotic enteric microbes (Cani et al., 2013; Galland, 2014). Although it is not surprising that this tenuous metabolic equilibrium between the eukaryotic host and its intestinal inhabitants is easily perturbed, it is essential that we understand and learn to control such perturbations because they might contribute greatly to the obesity and diabetes epidemics that are rampant in the developed world. Because of the ease with which the *Drosophila* model can be designed and manipulated, the accessibility of host genetic tools, and the simplicity of the fly genome and microbiota, this organism provides an ideal system in which to investigate the principles of the host interaction with its enteric microbial population.
